# Exosomal ORF3a mediates lung-liver axis to dysregulate hepatic lipid metabolism in mild COVID-19

**DOI:** 10.1038/s41421-026-00901-9

**Published:** 2026-05-23

**Authors:** Yafei Qu, Jinglin Zhou, Xinjia Wang, Shupeng Dong, Jingjiao Li, Lingxi Qiu, Lu Bian, Weiming Yuan, Qing Xie, Jieming Qu, Zhen Zhao, Xianfang Wu, Qiming Liang

**Affiliations:** 1 Center for Neurodegeneration and Regeneration, Zilkha Neurogenetic Institute, Department of Physiology and Neuroscience, Keck School of Medicine, University of Southern California, Los Angeles, CA USA; 2 Institute of Pediatric Infection, Immunity, and Critical Care Medicine, Shanghai Children’s Hospital, Shanghai Jiao Tong University School of Medicine, Shanghai, China; 3 Shanghai Institute of Immunology, Department of Immunology and Microbiology, Key Laboratory of Cell Differentiation and Apoptosis of Chinese Ministry of Education, Shanghai Jiao Tong University School of Medicine, Shanghai, China; 4 Department of Infection Biology, Lerner Research Institute, Cleveland Clinic Foundation, Cleveland, OH USA; 5 Department of Infectious Diseases, Ruijin Hospital, Shanghai Jiao Tong University School of Medicine, Shanghai, China; 6 Department of Molecular Microbiology and Immunology, Keck School of Medicine, University of Southern California, Los Angeles, CA USA; 7 Department of Pulmonary and Critical Care Medicine, Institute of Respiratory Diseases, Ruijin Hospital, Shanghai Jiao Tong University School of Medicine, Shanghai, China; 8 Shanghai Key Laboratory of Emergency Prevention, Diagnosis and Treatment of Respiratory Infectious Diseases, Shanghai, China; 9 National Research Center for Translational Medicine at Shanghai, Shanghai, China; 10 Division of Biomedical Informatics & Genomics, John W. Deming Department of Medicine, Tulane University School of Medicine, New Orleans, LA USA; 11 Center for Immune-Related Diseases at Shanghai Institute of Immunology, Ruijin Hospital, Shanghai Jiao Tong University School of Medicine, Shanghai, China

**Keywords:** Immunology, Molecular biology

Dear Editor,

The severe acute respiratory syndrome coronavirus-2 (SARS-CoV-2), the causative agent of the coronavirus disease 2019 (COVID-19), triggered a global pandemic and continues to pose a major global health challenge. While SARS-CoV-2 primarily infects the respiratory tract, it can also damage other organs, including the liver, during both acute infection and long COVID^1^. Liver involvement significantly increases the severity and mortality rate of COVID-19^2^. Although studies have shown that the virus can directly infect the liver cells in severe or fatal cases, it typically remains confined to the lungs in mild infections^3^. Therefore, the mechanisms by which mild COVID-19 causes liver damage and metabolic disruption remain unclear.

Our study initially included 133 patients with mild SARS-CoV-2 infection (ages 14–95) and 63 SARS-CoV-2-negative patients admitted to Ruijin Hospital in Shanghai, China, in February 2024 (Supplementary Table S1). Individuals with pre-existing liver conditions or recent medication use were excluded. Among the COVID-19-positive patients, 70 individuals (52.63%) exhibited abnormal liver function test results (LFTs), including 27 cases (38.57%) with elevated alanine aminotransferase (ALT) levels, 50 cases (71.43%) with elevated aspartate aminotransferase (AST) levels, 28 cases (40%) with elevated gamma-glutamyl transferase (GGT) levels, 9 cases (12.86%) with elevated alkaline phosphatase (ALP) levels, and 7 cases (10%) with elevated total bilirubin (TBIL) levels (Supplementary Table S1). Compared to the negative controls, the levels of ALT, AST, and GGT were significantly elevated in COVID-19-positive patients, whereas ALP and TBIL levels did not show significant changes (Supplementary Fig. S1a–e), indicating that liver dysfunction is prevalent even in mild COVID-19 cases.

We next investigated whether mild SARS-CoV-2 infection could induce liver injury in mice. To simulate mild COVID-19 infection, we utilized a mouse-adapted strain of SARS-CoV-2 (MA10) to infect wild-type C57BL/6J mice (Fig. 1a). MA10 induces acute lung-specific infection that closely resembles human SARS-CoV-2 infection^4^. Histological examination of the infected mice revealed that the expression of the nucleocapsid protein, indicative of active SARS-CoV-2 replication, was restricted to the respiratory tract and absent in liver tissue (Supplementary Fig. S2a). Consistently, quantitative RT-PCR analysis detected viral transcripts in the lung but not in the liver, suggesting that SARS-CoV-2 does not directly target the liver in this mild infection model (Supplementary Fig. S2b). Interestingly, in addition to its presence in the lungs, ORF3a was also detected in liver tissue (Fig. 1b). Beyond its predominant localization in hepatocytes (Fig. 1b), detailed confocal imaging revealed that ORF3a was also present in CD45-positive hematopoietic cells (Supplementary Fig. S2c). Notably, the overall level of lipid droplets (LDs) in the liver of MA10-infected mice was elevated compared to mock-infected controls (Fig. 1c, d). This increase is likely attributed to ORF3a-mediated inhibition of autophagosome-lysosome fusion or lysosome damage and dysfunction, resulting in LD accumulation^5–7^. Additionally, α-SMA staining indicated mild liver fibrosis in MA10-infected mice when compared to mock-infected controls (Fig. 1e, f). These findings demonstrate that ORF3a can access the liver and may induce abnormal liver metabolism in a mild COVID-19 model, even in the absence of detectable SARS-CoV-2 replication within the liver.

**Fig. 1:**
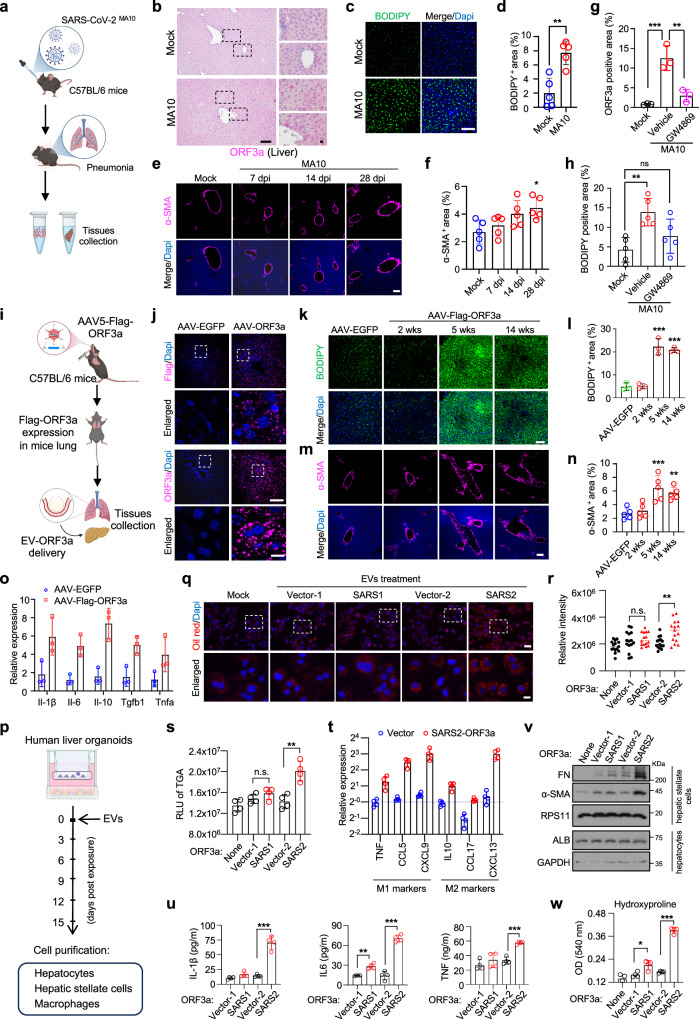
SARS-CoV-2 ORF3a activates a lung-liver axis to dysregulate liver lipid metabolism. **a** Schematic diagram illustrating the establishment of the mouse-adapted SARS-CoV-2 (MA10) infection model in C57BL/6J mice and the subsequent collection of tissue samples. **b** Representative immunohistochemistry (IHC) images showing ORF3a expression in lungs and livers from mock- or MA10-infected mice at 3 days post-infection (dpi). Bars, 100 µm or 10 µm. **c**, **d** Representative immunofluorescence (IF) images (**c**) and quantification (**d**) of neutral lipid levels in the livers from mock- or MA10-infected mice at 14 dpi. Bar, 100 µm. **P < 0.01 by Student’s *t*-test. **e**, **f** Representative IF images (**e**) and quantification (**f**) of α-SMA in the livers from mock- or MA10-infected mice at indicated dpi. Bar, 100 µm. **g** Quantification of ORF3a levels in livers from DMSO- or GW4869-treated mock- or MA10-infected mice at 7 dpi. **h** Quantification of LD levels in livers from DMSO- or GW4869-treated mock- or MA10-infected mice at 7 dpi. **i** Schematic diagram illustrating AAV5-mediated lung-specific expression model for EGFP or ORF3a. C57BL/6J mice were intratracheally infected with AAV5-EGFP or AAV5-Flag-ORF3a, after which EV-mediated ORF3a crosstalk occurred between the lung and liver. **j** Representative IF images showing Flag or ORF3a expression levels in livers from AAV5-EGFP- or AAV5-Flag-ORF3a-infected mice at 5 weeks post-infection. Bars, 50 µm or 10 µm. **k**, **l** Representative IF images (**k**) and quantification (**l**) of neutral lipid levels in the livers from AAV5-EGFP- or AAV5-Flag-ORF3a-infected mice at indicated weeks post-infection. Bar, 50 µm. **m**, **n** Representative IF images (**m**) and quantification (**n**) of α-SMA levels in the livers from AAV5-EGFP- or AAV5-Flag-ORF3a-infected mice at indicated weeks post-infection. Bar, 100 µm. **o** Relative mRNA levels of selected cytokines assessing inflammatory responses in the livers from AAV5-EGFP- or AAV5-Flag-ORF3a-infected mice at 6 weeks post-infection. **p** Schematic diagram illustrating the treatment of human liver organoids with purified EVs containing SARS-CoV or SARS-CoV-2 ORF3a (EV-ORF3a) or vector control (EV-vector) and subsequent analysis for liver damages. **q**, **r** Representative IF images (**q**) and quantification (**r**) of neutral lipid levels in hepatocytes at 15 days post-treatment with EVs containing vector control, SARS-CoV (SARS1) ORF3a, or SARS-CoV-2 (SARS2) ORF3a. Bars, 40 µm or 10 µm. **s** Relative levels of triglyceride in hepatocytes at 15 days post-treatment with EVs containing vector control, SARS1 ORF3a, or SARS2 ORF3a. **t** Relative mRNA levels of indicated M1 and M2 polarization markers in macrophages at 15 days post-treatment with EVs containing vector control or SARS2 ORF3a. **u** Protein levels of secreted inflammatory cytokines by macrophages at 15 days post-treatment with EVs containing vector control, SARS1 ORF3a, or SARS2 ORF3a. **v** The immunoblot results with indicated antibodies showing the activation of HSCs at 15 days post-treatment with EVs containing vector control, SARS1 ORF3a, or SARS2 ORF3a. **w** The hydroxyproline levels in HSCs at 15 days post-treatment with EVs containing vector control, SARS1 ORF3a, or SARS2 ORF3a. *P < 0.05, **P < 0.01, and ***P < 0.001 by one-way ANOVA with Tukey’s post hoc test in (**f**–**h**, **l**, **n**, **r**, **s**, **u**, **w**).

ORF3a is a small transmembrane protein that plays a critical role in the SARS-CoV-2 life cycle. Deletion of ORF3a from the SARS-CoV-2 genome significantly impairs viral replication and pathogenesis^8^. During infection, ORF3a can be efficiently secreted via exosomes by infected cells in vitro (Supplementary Fig. S3a). Therefore, we hypothesized that ORF3a-enriched exosomes are captured by the liver, leading to the accumulation of LDs and subsequent hepatic inflammation during mild COVID-19. Indeed, inhibiting exosome secretion by GW4869 reduced ORF3a levels in the liver of MA10-infected mice (Fig. 1g and Supplementary Fig. S3b). Hepatic LD accumulation and immune cell infiltration were subsequently reduced following GW4869 treatment in MA10-infected mice (Fig. 1h and Supplementary Fig. S3c–g). These findings indicate that ORF3a is transported to the liver via exosomes, where it disrupts liver lipid metabolism and triggers liver fibrosis in a mild COVID-19 model.

SARS-CoV-2 infection typically triggers an inflammatory cytokine storm, which can impact the extra-pulmonary organs due to hyperactivated immune responses. To eliminate the effects of the cytokine storm in our model, we established an AAV5-mediated lung infection model to introduce ORF3a expression in the lung, mimicking SARS-CoV-2 infection^9^. In this model, C57BL/6J mice were intratracheally intubated with either AAV5-ORF3a or AAV5-EGFP control virus (1 × 10^11^ vg), and tissues were harvested at 14 days post-infection (dpi) (Fig. 1i). While EGFP expression was only detected in the lungs, ORF3a was observed in both the lung and liver at 14 dpi, as confirmed by immunostaining and immunoblot analysis, reflecting the spreading property seen in MA10-infected mice (Fig. 1j and Supplementary Fig. S4a). Moreover, liver LD levels, visualized by BODIPY staining, were markedly elevated at 56 dpi in AAV5-ORF3a-infected mice compared to AAV5-EGFP controls (Fig. 1k, l). Additionally, the levels of neutral fats and lipids were significantly higher in the liver of AAV5-ORF3a-infected mice as shown by Oil Red O staining, indicating abnormal lipid accumulation in the liver in response to ORF3a expression in the lung (Supplementary Fig. S4b, c). The AAV5-mediated lung expression of ORF3a also enhanced PPARγ mRNA levels, suggesting dysregulation of hepatic lipid metabolism (Supplementary Fig. S4d). Lipid accumulation in hepatocytes can induce fibrogenic activation of hepatic stellate cells. Similar to mild SARS-CoV-2 infection in mice, AAV5-mediated ORF3a expression in the lung was sufficient to elevate α-SMA levels in the liver compared to the AAV5-EGFP controls (Fig. 1m, n), indicating that ORF3a communication through the lung-liver axis results in hepatic stellate cell activation and liver fibrosis. Additionally, the population of CD68-positive Kupffer cells was significantly increased in AAV5-ORF3a-infected mice relative to the AAV5-EGFP control group (Supplementary Fig. S4e, f). The mRNA levels of inflammatory cytokines, including IL-6, IL-1β, IL-10, TGFβ, and TNFα, were also significantly elevated due to ORF3a communication via lung-liver axis compared to the control EGFP group (Fig. 1o). These findings demonstrate that ORF3a-mediated lung-liver axis is sufficient to disrupt lipid metabolism and cause inflammation in the liver.

To further investigate the impact of ORF3a via exosomes on the human liver system, we utilized a human pluripotent stem cell (hPSC)-derived multicellular liver culture model that mimics the composition of the human liver and recapitulates the functional characteristics of hepatocytes, enabling the study of human liver lipid metabolism in the presence of exosomal ORF3a in vitro^10,11^. We individually differentiated hPSCs to hepatocytes (hPSC-hepatocytes), hepatic stellate cells (hPSC-HSCs), and macrophages (hPSC-macrophages) and co-cultured hPSC-hepatocytes, hPSC-HSCs, and hPSC-macrophages at an 8:1:1 ratio in a transwell culture system, allowing for the separation of macrophages from the co-cultured hepatocytes and HSCs while facilitating the diffusion of secreted factors (Fig. 1p). In this culture environment, clusters of 3–6 HSCs surrounded by numerous hepatocytes were formed, while macrophages adhered to the transwell membrane, maintaining stability for at least three weeks without proliferation, as we previously reported^10,11^. Subsequently, exosomes loaded with ORF3a (EV-ORF3a) or control vector (EV-Vector) were introduced into the culture medium, and the effects on LD metabolism and associated cellular damage were assessed at 3 days post-treatment (Fig. 1p). The delivery of EV-ORF3a did not affect the cell viability or albumin secretion (Supplementary Fig. S5a, b), but led to increased lipid accumulation and elevated triacylglycerol (TAG) levels in hepatocytes compared to EV-Vector treatment (Fig. 1q–s). The levels of lipogenesis-associated enzymes, including FASN, ACACA, and SCD, were also significantly upregulated in hepatocytes upon EV-ORF3a treatment (Supplementary Fig. S5c–e), suggesting that exosomal ORF3a dysregulates lipid metabolism in human multicellular liver culture. Additionally, EV-ORF3a treatment led to the induction of both M1 markers (TNFα, CCL5, and CXCL9) and M2 markers (IL-10, CCL17, and CXCL13) compared to the control EV-Vector treatment in the human organoid models (Fig. 1t). The inflammatory cytokines, including IL-1β, IL-6, and TNFα, were significantly elevated in macrophages upon EV-ORF3a treatment, as shown by quantitative RT-PCR in macrophages and ELISA using culture supernatant (Fig. 1u and Supplementary Fig. S5f), suggesting that exosomal ORF3a activates liver macrophages and triggers liver inflammation. Moreover, EV-ORF3a, but not EV-Vector, promoted the differentiation of quiescent HSCs into activated myofibroblasts expressing high levels of α-SMA and hydroxyproline (Fig. 1v, w), indicating that exosomal ORF3a causes the activation of HSCs and subsequent liver fibrosis. These findings demonstrate that SARS-CoV-2 ORF3a disrupts human liver lipid metabolism and induces liver inflammation, partly resembling key features of nonalcoholic fatty liver disease.

Previous research from our team has revealed that SARS-CoV-2 ORF3a induces the accumulation of cellular LD by interacting with Vps39 from the HOPS complex and obstructing the fusion process between autophagosomes and lysosomes^12^. Despite sharing 72.7% amino acid identity with ORF3a^SARS-CoV-2^, ORF3a^SARS-CoV^ does not engage with Vps39 or provoke LD accumulation^5,6,12^. In line with these findings, ORF3a^SARS-CoV^ is unable to alter liver lipid metabolism in the hPSC-derived multicellular liver culture system to the same extent as ORF3a^SARS-CoV-2^, as evidenced by the lipid buildup in hepatocytes, the levels of inflammatory cytokines secreted by macrophages, and activation of HSCs (Fig. 1q–s, u–w and Supplementary Fig. S5c–f). These results suggest that SARS-CoV-2 exerts its specific influence on liver lipid metabolism with ORF3a by targeting the HOPS complex subunit Vps39.

In summary, our study demonstrates that during mild COVID-19, the SARS-CoV-2 ORF3a travels from the lungs to the liver via exosomes, establishing a pathogenic lung-liver axis (Supplementary Fig. S6). In the liver, ORF3a disrupts lipid metabolism by preventing the autophagic breakdown of LDs, leading to fat accumulation and inflammation. This lipid-disrupting ability is conserved across most major SARS-CoV-2 variants. However, it is absent in the Beta variant due to a specific mutation (S171L) that prevents ORF3a from binding to the host protein Vps39 (Supplementary Fig. S7)^12^. Since the liver clears over 90% of circulating exosomes, it acts as a primary target for lung-derived, ORF3a-carrying exosomes^13^. By hijacking this exosome transport system, ORF3a can damage distal organs like the liver even when the virus remains confined to the respiratory tract. Therefore, targeting ORF3a-mediated lung-liver axis with specific inhibitors or antibodies presents a promising therapeutic strategy for mitigating COVID-19-related liver damage.

## Supplementary information

Supplemental Materials
