# Supplementary material for: Exosomal ORF3a mediates lung-liver axis to dysregulate hepatic lipid metabolism in mild COVID-19
Source: Cell Discov. 2026 May 23;12:36. doi: 10.1038/s41421-026-00901-9 (PMC13198539; doi:10.1038/s41421-026-00901-9)
Supplement: Supplementary file 1 — Supplemental Materials [file 41421_2026_901_MOESM1_ESM.pdf]

Supplementary Information

Supplementary Table S1. Mild COVID-19 patients show abnormal liver function test results

Characteristics	Health individuals (n=63)	Mild COVID (n=132)	P value
Age (years), mean ± SD	60.51 ± 20.50	62.71 ± 17.90	0.444
Male gender, n (%)	34 (54.0)	77 (58.3)	0.674
ALT (U/L), median [IQR]	16.0 [10.0-25.0]	19.5 [12.2-37.8]	0.024
Normal	60 (95)	101 (77)	
1-2 ULN, n (%)	3 (4.8)	22 (17)	
2-3 ULN, n (%)	0 (0)	4 (3.0)	
>3 ULN, n (%)	0 (0)	5 (3.8)	
AST (U/L), median [IQR]	20.0 [16.0-27.0]	29.0 [20.0-46.0]	<0.001
Normal	57 (90)	77 (58)	
1-2 ULN, n (%)	6 (9.5)	42 (32)	
2-3 ULN, n (%)	0 (0)	7 (5.3)	
>3 ULN, n (%)	0 (0)	6 (4.5)	
ALP (U/L), median [IQR]	69.0 [58.0-79.0]	70.0 [57.0-87.8]	0.6641
Normal	61 (97)	122 (92)	
1-2 ULN, n (%)	2 (3.2)	10 (7.6)	
2-3 ULN, n (%)	0 (0)	0 (0)	
>3 ULN, n (%)	0 (0)	0 (0)	
GGT (U/L), median [IQR]	19.0 [12.0-25.0]	23.0 [15.0-44.0]	0.0067
Normal	58 (92)	100 (76)	
1-2 ULN, n (%)	3 (4.8)	25 (19)	
2-3 ULN, n (%)	1 (1.6)	5 (3.8)	
>3 ULN, n (%)	1 (1.6)	2 (1.5)	
TBIL (μmol/L), median [IQR]	10.6 [7.6-13.6]	10.8 [7.7-16.8]	0.4941
Normal	62 (98)	124 (94)	
1-2 ULN, n (%)	1 (1.6)	6 (4.5)	
2-3 ULN, n (%)	0 (0)	1 (0.8)	
>3 ULN, n (%)	0 (0)	1 (0.8)	

ALT, alanine aminotransferase; AST, aspartate aminotransferase; ALP, alkaline phosphatase; GGT, gamma-glutamyltransferase; TBIL, total bilirubin; ULN, upper limit of normal. Categorical variables were presented as frequencies and percentage. Continuous variables were expressed as mean ± SD or median [IQR]. Judgment criteria: ALT>40 IU/L, AST>35 IU/L, ALP>125 IU/L, GGT>45 IU/L, TBIL>24 μmol/L.

Supplementary Fig. S1. Mild COVID-19 patients show abnormal liver function test results

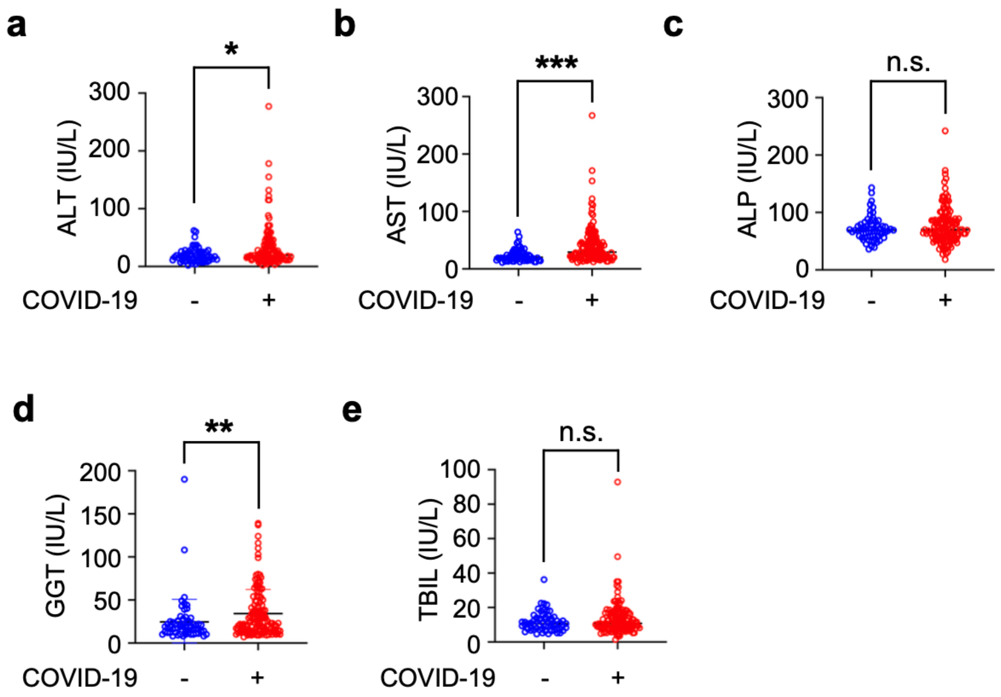

The levels of liver function markers, including ALT (**a**), AST (**b**), ALP (**c**), GGT (**d**), and TBIL (**e**) in mild COVID-19 patients and healthy individuals. n=63 for health individuals; n=132 for mild COVID-19 group. n.s., not significant; **P*<0.05; ***P*<0.01; ****P*<0.001 by Mann–Whitney U test.

Supplementary Fig. S2. ORF3a enters liver during mild COVID-19 infection

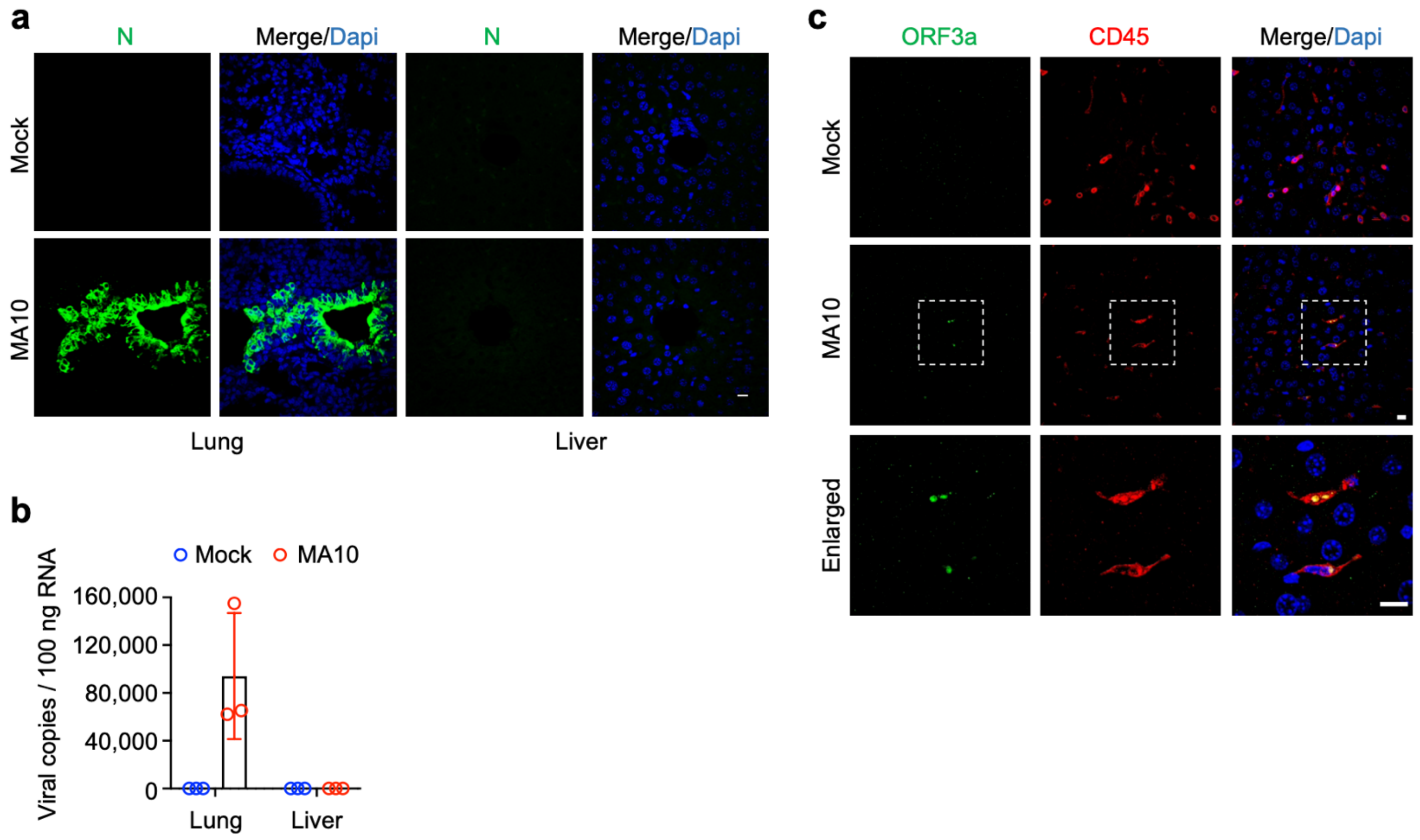

(**a**) Representative immunofluorescence (IF) images of nucleocapsid (N) levels in lungs and livers from mock- or MA10-infected mice at 3 dpi. Bar=10 μm. (**b**) Viral genome copies in both lung and liver tissues from mock- or MA10-infected mice were quantified by qRT-PCR at 3 dpi. n=3. (**c**) Representative IF images of ORF3a (green) and CD45 (red) in the liver from mock- or MA10-infected mice at 7 dpi. The boxed areas are shown at higher magnification in the panels below. Bars=10 μm.

Supplementary Fig. S3. Exosomes mediate ORF3a lung-liver crosstalk during mild COVID-19 infection

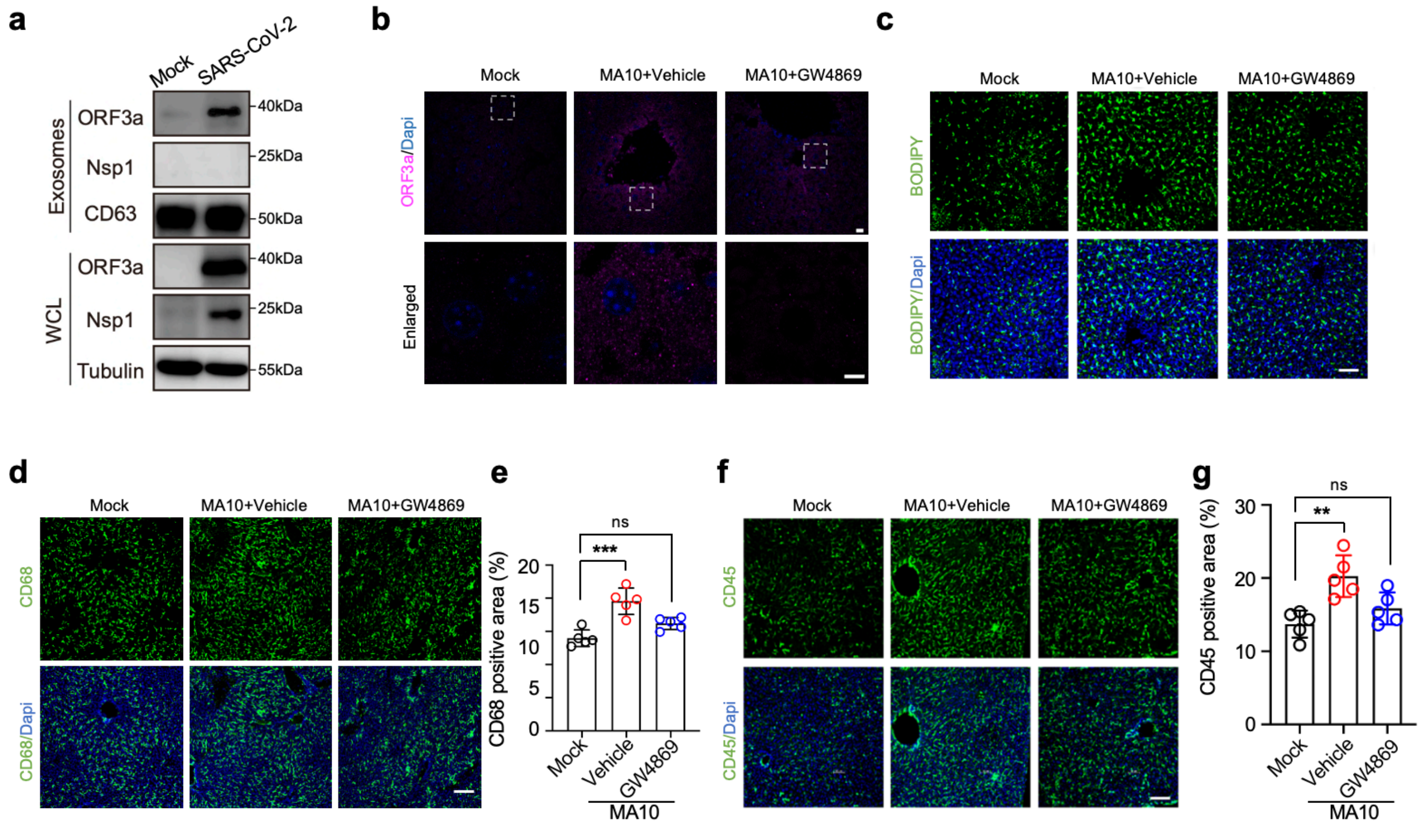

(**a**) EVs purified from mock- or SARS-CoV-2-infected Vero cells (MOI: 0.1) at 48 hours post-infection (hpi) were subjected to immunoblot (IB) with indicated antibodies. (**b**) Representative IF images of ORF3a in livers from mock- or MA10-infected mice treated with DMSO or GW4869 (2.5 μg/g body weight) at 7 dpi. Bars=10 μm or 5 μm. (**c**) Representative IF images of LDs in livers from mock- or MA10-infected mice treated with DMSO or GW4869 at 7 dpi. Bar=50 μm. (**d-e**) Representative IF images (d) or quantification (e) of CD68 in livers from mock- or MA10-infected mice treated with DMSO or GW4869 at 7 dpi. Bar=50 μm. n=5. ***P<0.001 by one-way ANOVA with Tukey's post hoc test. (**f-g**) Representative IF images (f) or quantification (g) of CD45 in livers from mock- or MA10-infected mice treated with DMSO or GW4869 at 7 dpi. Bar=50 μm. n=5. **P<0.01 by one-way ANOVA with Tukey's post hoc test.

Supplementary Fig. S4. ORF3a mediates lung-liver axis is sufficient to dysregulate liver lipid metabolism

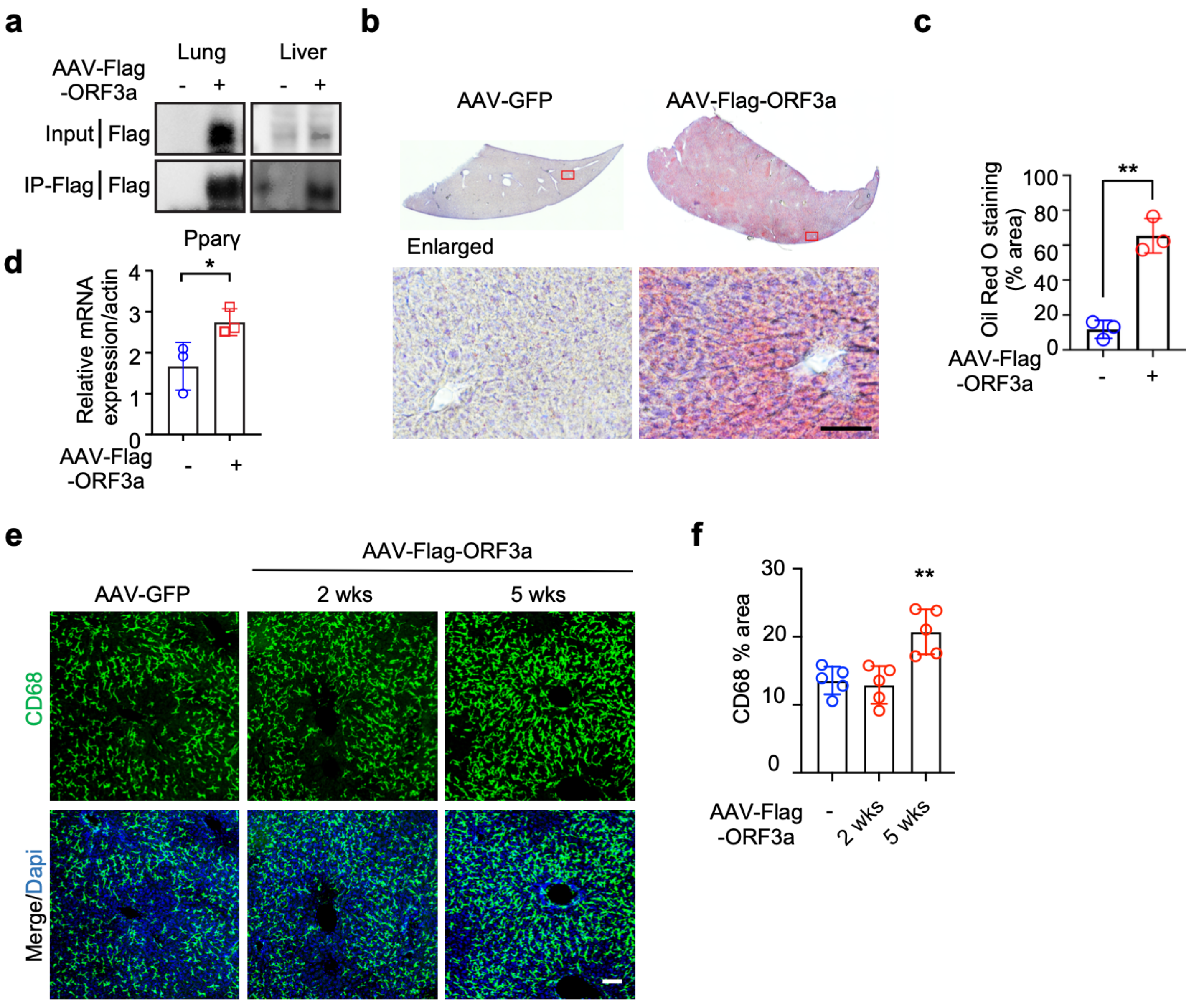

(**a**) IB analysis of ORF3a expression in lungs and livers from mice infected with AAV5-EGFP or AAV5-Flag-ORF3a at 14 dpi. (**b-c**) Representative Oil Red O staining (**b**) and quantification (**c**) in the livers from AAV5-EGFP- or AAV5-Flag-ORF3a-infected mice at 5 weeks post infection. The boxed areas are shown at higher magnification in the panels below. Bar=50 μm. n=3. ***P*<0.01 by Student's t-test. (**d**) Relative mRNA level of Pparγ in the liver tissues by qRT-PCR. Data are normalized to Actin. n=3. **P*<0.05 by Student's t-test. (**e-f**) Representative IF images (**e**) and quantification (**f**) of CD68 expression in the livers from AAV5-EGFP- or AAV5-Flag-ORF3a-infected mice at indicated weeks post infection. Bar=50 μm. n=5. ***P*<0.01 by one-way ANOVA with Tukey's post hoc test.

Supplementary Fig. S5. Exosomal ORF3a$^{\text{SARS-CoV-2}}$ dysregulates lipid metabolism in human liver organoids

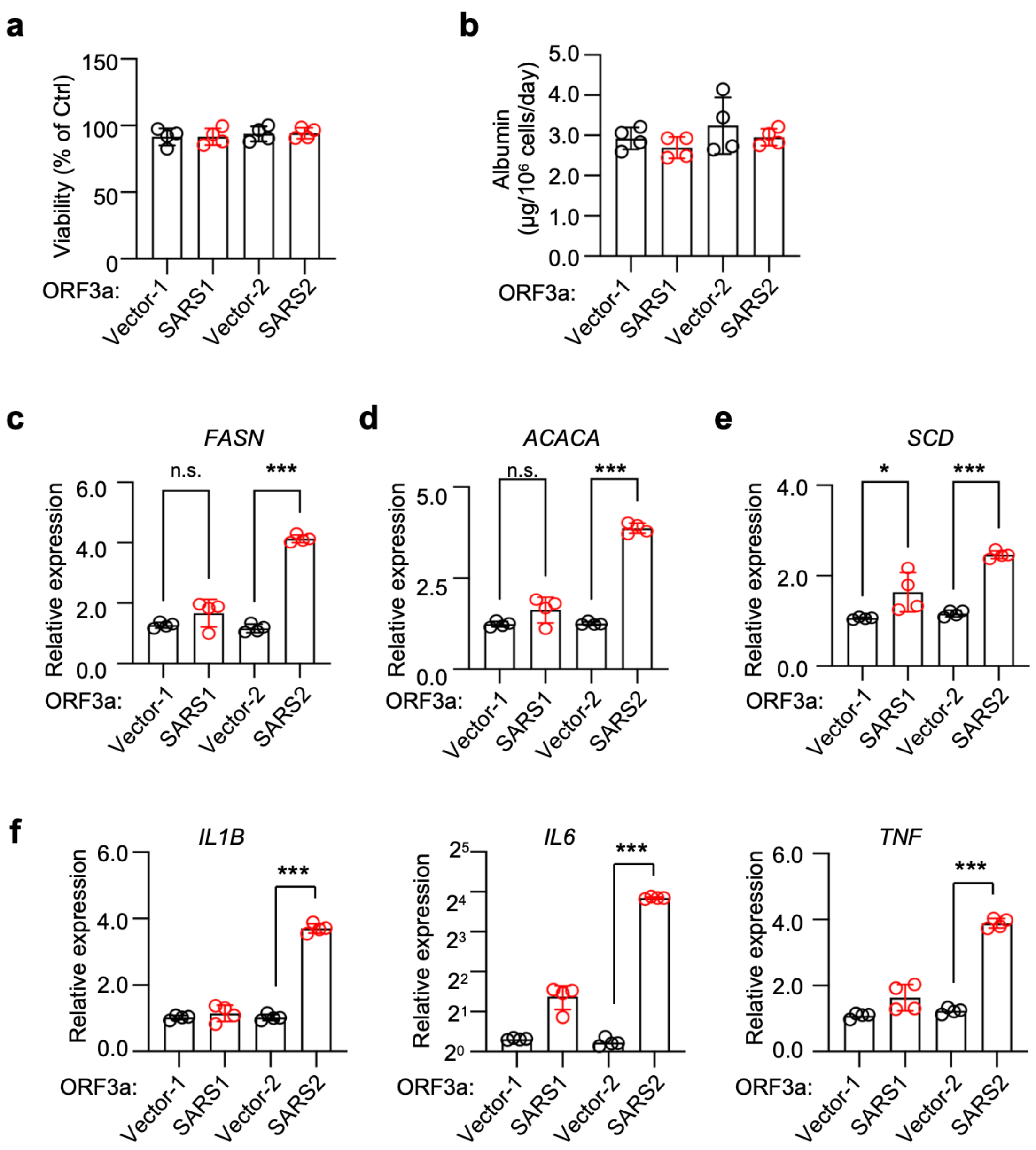

(**a-b**) Exosomal ORF3a does not affect cell viability (**a**) and albumin secretion (**b**) in human liver organoids. n=4. (**c-e**) The relative mRNA levels of FASN (**c**), ACACA (**d**), and SCD (**e**) in hepatocytes isolated from human liver organoids at 15 days post-treatment with EVs containing vector control, SARS1 ORF3a, or SARS2 ORF3a. n=4. n.s., not significant; **P*<0.05; ****P*<0.001 by one-way ANOVA with Tukey's post hoc test. (**f**) Relative mRNA levels of inflammatory cytokines in macrophages at 15 days post-treatment with EVs containing vector control, SARS1 ORF3a, or SARS2 ORF3a. n=4. ****P*<0.001 by one-way ANOVA with Tukey's post hoc test.

Supplementary Fig. S6. Schematic diagram illustrating that ORF3a activates lung-liver axis via exosome to dysregulate hepatic lipid metabolism in mild COVID-19

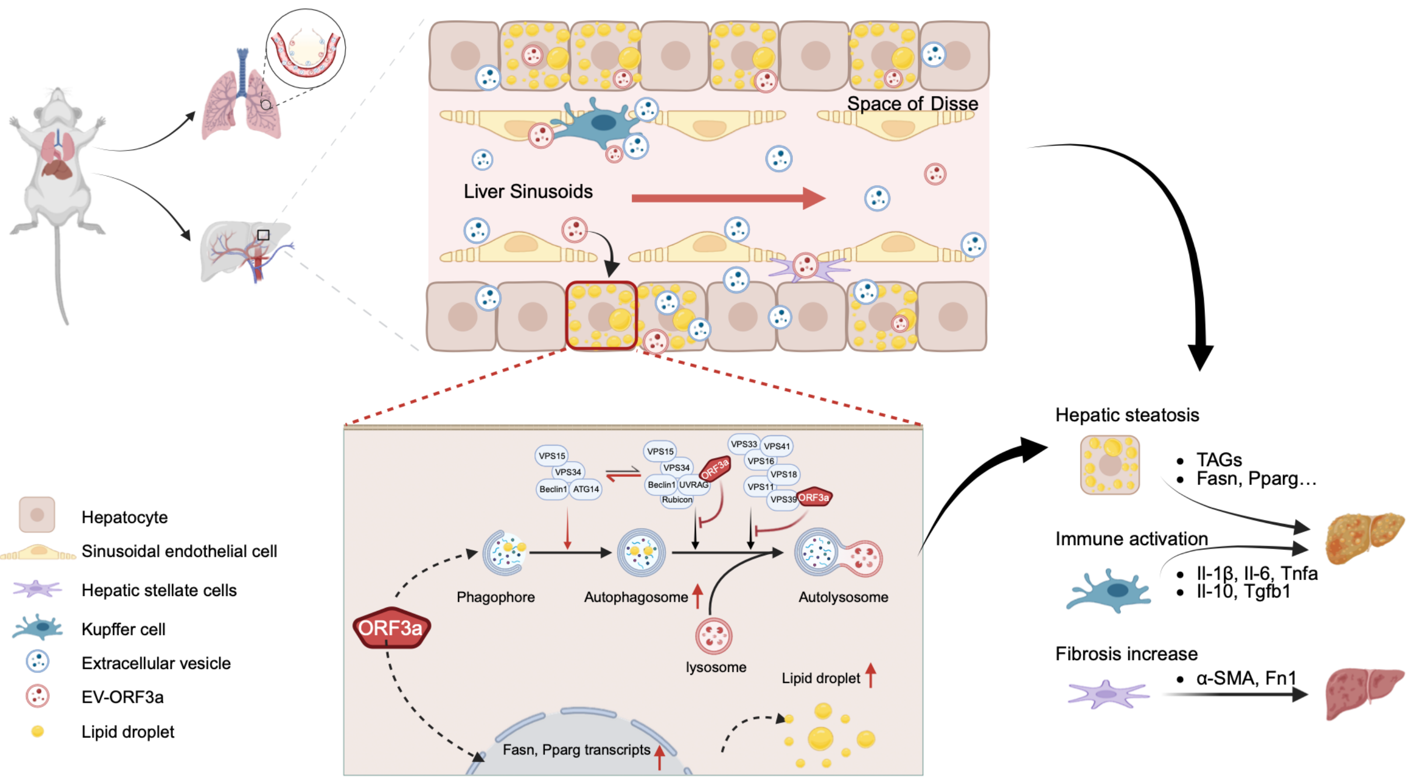

Supplementary Fig. S7. ORF3a^{S171L} does not cause lipid accumulation and liver dysfunction.

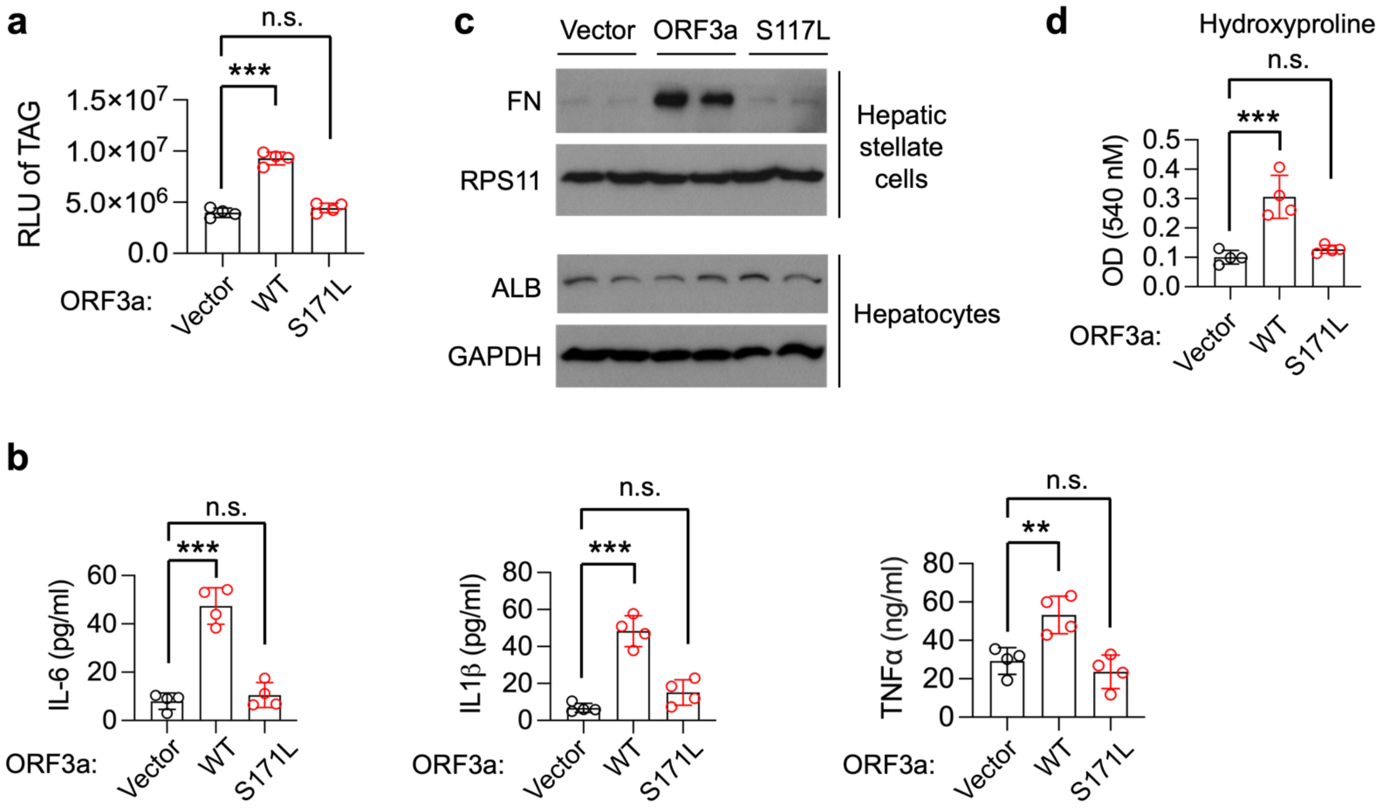

(**a**) Relative levels of TGA in hepatocytes at 15 days post-treatment with EVs containing vector control, SARS2 ORF3a, or SARS2 ORF3a^{S171L} mutant. n.s. and ****P*<0.001 by one-way ANOVA with Tukey's post hoc test. (**b**) Protein levels of secreted inflammatory cytokines by macrophages at 15 days post-treatment with EVs containing vector control, SARS2 ORF3a, or SARS2 ORF3a^{S171L} mutant. ns, ***P*<0.01 and ****P*<0.001 by one-way ANOVA with Tukey's post hoc test. (**c**) The immunoblot results with indicated antibodies showing the activation of hepatic stellate cells (HSCs) at 15 days post-treatment with EVs containing vector control, SARS2 ORF3a, or SARS2 ORF3a^{S171L} mutant. (**d**) The hydroproline levels in HSCs at 15 days post-treatment with EVs containing vector control, SARS2 ORF3a, or SARS2 ORF3a^{S171L} mutant. n.s. and ****P*<0.001 by one-way ANOVA with Tukey's post hoc test.

Materials and Methods

Viruses, Plasmids, and Cells

Mouse-adapted SARS-CoV-2 MA10 was obtained from BEI Resources (#NR-55329). SARS-CoV-2 MA10 stocks were propagated in Vero-E6 cells, and the titer of MA10 stocks was determined by standard plaque assay on Vero-E6 cells. AAV5-EGFP and AAV5-Flag-ORF3a were purchased from GenePharma (Shanghai, China).

ORF3a from SARS-CoV-2 or SARS-CoV was synthesized by GenScript Biotech and cloned into pLVX or pEF vectors as indicated. All constructs were sequenced using an ABI PRISM 377 automatic DNA sequencer to verify 100% correspondence with the original sequence.

HEK293T cells (ATCC, #CRL-11268) were maintained in Dulbecco's modified Eagle's medium (DMEM; Gibco, #11965092) containing 10% Fetal Bovine Serum (FBS; Corning, #35-015-CV) and 1% penicillin-streptomycin (Gibco, #15140122) at 37°C with 5% CO_2. Human pluripotent stem cells (hPSCs: iPSC-W3) were maintained on growth factor-reduced Matrigel in feeder-independent mTeSR1-based medium, either regular mTeSR1 (Stemcell Technologies, #85850) or mTeSR1 Plus (Stemcell Technologies, #100-0276) according to the manufacturer's instructions. Culture medium was refreshed daily, and cells were passaged every 4-6 days as clumps using ReLeSR (Stemcell Technologies, #05872). For all the experiments in this study, iPSCs were used between passage 30 and 40. Transient transfections were performed with Lipofectamine 3000 (Thermo Fisher Scientific, #3000015) according to the manufacturer's instruction.

Animal models

In this study, 12-week-old C57BL/6J mice (Jackson Laboratory, #000664) were used to establish a SARS-CoV-2 MA10 infection model as reported.[16] Mice were anesthetized with isoflurane and intranasally inoculated with either 50 µL phosphate-buffered saline (PBS; pH 7.4) or 1×10^4 PFU of MA10 diluted in 50 µL PBS (per mouse). Following infection, mice were monitored daily and euthanized under deep isoflurane anesthesia at the indicated time points. Brain, lung, and liver tissues were harvested and either stored in TRIzol (Sigma-Aldrich, #T9424) at –80°C freezer or fixed in 4% paraformaldehyde (PFA) at 4°C until further analyses.

To establish an AAV5-mediated lung infection model, 12-week-old C57BL/6J mice were anesthetized with isoflurane and intratracheally intubated with either AAV5-Flag-ORF3a or AAV5-EGFP control virus (1×10^{11} vg per mouse). Tissues were harvested at the indicated days post-infection as described above.

All animal experiments were conducted in accordance with the National Institutes of Health Guide for the Care and Use of Laboratory Animals and were approved by the Institutional Animal Care and Use Committees (IACUC). Experiments involving infectious virus (MA10) were performed in a certified animal biological safety level 3 (ABSL-3) facility, whereas AAV5-mediated experiments were conducted under animal biological safety level 2 (ABSL-2) facility at the University of Southern California Keck School of Medicine.

Immunohistochemical staining

For immunohistochemistry, 8-µm paraffin-embedded liver sections were deparaffinized and rehydrated through xylenes and a graded ethanol series (100%, 90%, 70%, 50%), followed by rinsing in ddH_2O. Antigen retrieval was performed by incubating sections in a citric acid-based antigen unmasking solution (Vector Laboratories, #H-3300) at 80°C for 15 min. IHC staining was carried out using the ImmPRESS Duet Double Staining HRP/AP Polymer Kits (Vector Laboratories, #MP-7724) according to the manufacturer's instructions. Briefly, sections were blocked and incubated with anti-ORF3a antibody (Cell Signaling Technology, #34340, 1:100), followed by incubation with the ImmPRESS Dual HRP/AP Polymer Reagent. Signals were developed using ImmPACT DAB EqV Substrate (AP, magenta) for 20-30 min. Sections were then counterstained with hematoxylin (Vector Laboratories, #H-3502). Oil Red O (Thermo Scientific, #A12989-22) staining was performed following manufacturer's instructions. Images were acquired using a Nikon Ti2 inverted microscope equipped with a digital camera and processed using ImageJ software.

Immunofluorescence

8-µm paraffin-embedded liver sections, prepared as described above, or vibratome sections were washed with PBS and blocked in 5% bovine serum albumin (BSA) in PBST (0.3% Triton-X 100 in PBS) for 30 min at room temperature (RT). Tissue sections were incubated with indicated primary antibodies overnight at 4°C, followed

by incubation with secondary antibodies for 1 h at RT. Hoechst 33342 (Thermo Scientific, #62249) was used for nuclear counterstaining. Primary antibodies used in this study included anti-α-SMA (Cell Signaling Technology, #19245), anti-Flag (Cell Signaling Technology, #14793), anti-SARS-CoV-2 ORF3a (Cell Signaling Technology, #34340), anti-SARS-CoV-2 ORF3a (Sicgen Antibodies, #SCG3-AB0408), anti-SARS-CoV-2 N (GeneTex, #HL344), anti-CD45 (Santa Cruz Biotechnology, #sc-53665), and anti-CD68 (Thermo Fisher Scientific, #14-0681-82). All secondary antibodies used in this study were purchased from Thermo Fisher Scientific. BODIPY (4,4-Difluoro-1,3,5,7,8-Pentamethyl-4-Bora-3a,4a-Diaza-s-Indacene; Thermo Fisher Scientific, #D3922) and Oil Red O (Thermo Scientific, #A12989-22) staining were performed according to the manufacturer's instructions. Images were acquired using a Nikon Ti2 confocal microscope equipped with an automated stage and were further analyzed using ImageJ software. Identical laser intensity, detector sensitivity, gain, and offset settings were applied to all images within each experiment.

Immunoprecipitation and Immunoblot Analysis

For tissue immunoprecipitation assays, mice infected with AAV5-EGFP or AAV5-Flag-ORF3a were euthanized, and tissues were harvested and lysed using Tissue Protein Extraction Reagent (Thermo Fisher Scientific, #78510) supplemented with Protease Inhibitor Cocktail (Thermo Fisher Scientific, #78429), according to the manufacturer's instructions. Tissue samples were incubated on ice for 30 min and subsequently sonicated at 30% amplitude for 30 s (10 s ON, 10 s OFF). Lysates were clarified by centrifugation and incubated with anti-Flag M2 Magnetic Beads (Sigma-Aldrich, #M8823) at 4°C overnight. Following incubation, beads were washed and eluted with 4X Bolt LDS Sample Buffer (Thermo Fisher Scientific, #B0007). Samples were resolved by SDS-PAGE and transferred onto PVDF membrane (Bio-Rad). Membranes were blocked with 5% nonfat milk in PBST (0.05% Tween-20 in PBS) and incubated with the indicated primary antibodies diluted in 5% BSA at 4°C overnight. Primary antibodies used in this study included anti-Flag (Cell Signaling Technology, #14793), anti-SARS-CoV-2 ORF3a Antibody (ABClonal, #A20234), anti-SARS-CoV-2 Nsp1 Antibody (GeneTex, #GTX135612), anti-CD63 (Thermo Fisher Scientific, #446703), anti-β-Tubulin (Proteintech, #10094), anti-FN (GeneTex, #GTX112794), anti-α-SMA (Cell Signaling Technology, #19245), anti-RPS11 (Bethyl Laboratories, #A303-936A-T), and anti-ALB (GeneTex, #GTX102419). HRP-conjugated secondary antibodies were applied in 5% nonfat milk, and protein bands were visualized using enhanced chemiluminescence (ECL; Thermo Scientific, A38555) and imaged on a Fuji LAS-4000 imager.

Exosome purification

HEK293T cells were seeded in 10-cm culture dishes and transfected at 80-90% confluency with 20 µg of vector or ORF3a expression plasmids (from either SARS1 or SARS2) using 60 µL polyethyleneimine (PEI; YEASEN, #40820). Transfected cells were cultured for 48 h, after which exosomes were isolated from the culture medium using the exoEasy Maxi Kit (QIAGEN, #76064) according to the manufacturer's instructions.

Serum exosome purification

Blood samples were collected from mock- or SARS-CoV-2 MA10-infected mice at 5-7 dpi by retro-orbital bleeding using heparinized glass capillary tubes under isoflurane anesthesia. Serum was isolated according to standard procedures, and exosomes were purified using the Total Exosome Isolation Reagent (from serum) (Invitrogen, #4478360) following the manufacturer's instructions.

Flow cytometry analysis of exosomes

Serum-derived exosomes were fixed by 2% PFA for 30 minutes and were subsequently incubated with anti-CD63 (Thermo Fisher Scientific, #446703) and anti-ORF3a (Sicgen Antibodies, #SCG3-AB0408) antibodies followed by incubation with appropriate secondary antibodies. Labeled EVs were washed with PBS by ultracentrifugation at 100,000 x g for 2 h and resuspended in 100 µL PBS prior to analysis on a BD FACSAria II Flow Cytometer (BD Biosciences, USA).

Differentiation of hPSC-derived hepatic lineages

Human pluripotent stem cells (hPSCs: iPSC-W3) were differentiated into hepatic stellate cells (HSCs), hepatocytes (HEPs), and macrophages as described below.

Differentiation of quiescent HSCs. Cell culture plates were coated twice with growth factor-reduced Matrigel to increase the matrix thickness. hPSCs were dissociated into a single-cell suspension and seeded onto a

Matrigel-coated plate in mTeSR1 medium supplemented with 10 µM ROCK inhibitor (Y-27632; Stemcell Technologies, #72308) to reach approximately 20% confluence by the next day. Mesoendoderm differentiation was initiated by culturing hPSCs in RPMI 1640/B-27 medium (RPMI 1640, Life Technologies, #22400071; 2% B-27 minus insulin, Life Technologies, # A1895601; 0.5% GlutaMax, Life Technologies, #35050-061; 0.5% non-essential amino acid, Life Technologies, #11140050) containing 10 µM CHIR99021 (Stemcell Technologies, #72054) for one day, followed by RPMI/B-27 with 20 ng/mL BMP4 (Peprotech, #AF-120-05ET) for three days to promote mesoderm generation. The mesodermal progenitors were subsequently grown in RPMI/B-27 medium supplemented with 50 µg/mL of ascorbic acid (Sigma-Aldrich, #A4544), 0.5% ITS (Life Technologies, #41400-045), 0.5 µM dexamethasone (Sigma-Aldrich, #D4902), 5 ng/mL BMP4, and 20 ng/mL FGF1 (Peprotech, #100-17A) for three days. Quiescent HSCs were finally induced by culturing cells in RPMI/B-27 supplemented with 50 µg/mL of ascorbic acid (Sigma-Aldrich, #A4544), 0.5% ITS (Life Technologies, #41400-045), 0.5 µM dexamethasone (Sigma-Aldrich, #D4902), 1% synthetic lipids (Sigma-Aldrich, #L0288), 30 ng/mL EGF (Peprotech, #AF-100-15), 10 ng/mL FGF2 (Life Technologies, #PHG0024), and 5 µM retinol (Sigma-Aldrich, #R7632) for six days.

Differentiation of HEPs. hPSCs were seeded onto Matrigel-coated plates as described above and differentiated into definitive endoderm cells using the STEMdiff Definitive Endoderm Kit (Stemcell Technologies, #05110) according to the manufacturer's instructions. Definitive endoderm cells were then dissociated into single-cell suspensions using Accutase (Innovative Cell Technologies, #AT104-500) and plated onto Matrigel-coated plates in RPMI/B-27 medium supplemented with ROCK inhibitor, 20 ng/mL BMP4, and 10 ng/mL FGF2. The culture medium was refreshed daily for five days. Cells were then cultured in RPMI/B-27 containing 20 ng/mL HGF (Peprotech, #100-39) for five days, followed by further maturation in Lonza hepatocyte culture medium (Lonza, #CC-3198) supplemented with ascorbic acid, BSA-FAF, hydrocortisone, transferrin (Sigma-Aldrich, #T4132), insulin (Sigma-Aldrich, #91077C), GA-1000, and 20 ng/mL oncostatin M (OSM; R&D systems, #295-OM-050) for one to two weeks. Culture medium was replaced every two days.

Differentiation of macrophages. hPSCs were first differentiated into hematopoietic stem cells using the Spin-EB method. Briefly, 3000 hPSC cells were seeded into U-bottom 96-well non-tissue culture plates in 50 µL of serum-free medium (IMDM/Ham's F12 (1:1), Life Technologies, #12440046/#11765054, with 5 mg/mL BSA, 1x insulin-transferrin-selenium, 1x synthetic lipids, 50 µg/mL of ascorbic acid, and 2 mM GlutaMax), supplemented with 10 ng/mL BMP4, 10 ng/mL FGF2, and 10 µM Y-27632 for two days. Medium was further supplemented with 20 ng/mL VEGF (Peprotech, #100-20) every three days. On day 8, half of the culture medium was replaced with fresh medium containing 10 ng/mL FGF2, 10 ng/mL VEGF, and 50 ng/mL SCF (Peprotech, #300-07) until day 14. Hematopoietic stem cells were purified using the EasySep human CD34 positive selection kit (Stemcell Technologies) and expanded in StemSpan SFEM II supplemented with 10% CD34+ expansion supplement (Stemcell Technologies, #0269135050-061) for three days. Macrophage differentiation was initiated by culturing hematopoietic stem cells in induction medium (IMDM with 3% AB serum, Atlanta Bbiologicals, #S40110H; 2% human plasma, Stemcell Technologies, #70039; 10 ng/mL insulin; 3 U/mL heparin, Sigma-Aldrich, #H3149; 200 µg/mL transferrin) containing 50 ng/mL SCF, 1 ng/mL IL-3 (Peprotech, #200-03), 50 ng/mL Flt3 (Peprotech, #300-19), and 100 ng/mL M-CSF (Peprotech, #300-25) for 7-11 days. Medium was replenished every three days, and cultures were maintained at a cell density of approximately 1.0-1.5 million/mL.

Establishment of a multicellular co-culture system for human liver organoids

hPSC-derived HSCs, HEPs, and macrophages were differentiated separately as described above. HEPs at day 15 and HSCs at day 12 of differentiation were dissociated into a single-cell suspension using Accutase supplemented with 10 µM Y-27632. The dissociated HEPs and HSCs were mixed at a ratio of 8:1 and re-plated onto a Matrigel-coated 12-well cell culture plate at a density of 0.4 $\times 10^6$ cells per well in a mixed medium consisting of 50% final-stage HEP medium and 50% final-stage HSCs medium, supplemented with 10 µM Y-27632. On the following day, macrophages at day 9 of differentiation were added to the upper chamber of a transwell system at a 1:1 ratio with HSCs, and the transwell insert was placed into the wells containing HEPs/HSCs. The culture medium was changed to basal maintenance medium (BMM) which consisted of glucose-free DMEM (Life Technologies, #21063029) supplemented with 2% knockout serum replacement (KOSR; Life Technologies, #10828028), 2% B-27, 3 U/mL heparin, 200 µg/mL Transferrin, 30 ng/mL EGF, 5 µM retinol, and 0.5 µM dexamethasone. The 12-well plates containing the co-culture system were maintained on an orbital shaker platform at 30 rpm, and the culture medium was replenished every two days.

Cell viability measurement in human liver organoids
Cell viability was assessed using the CellTiter-Glo Luminescent Cell Viability Assay kit (Promega, #G7572) according to the manufacturer's instruction.

Separation of HEPs from HSCs
hPSC-derived co-cultures of HEPs and HSCs were first washed once with Versene (Life Technologies, #15040066) and incubated at 37°C for 20-25 min to loosen cell-cell contacts. Cells were then rinsed with pre-warmed DMEM/F12 and incubated in a pre-warmed collagenase mixture (2 mg/mL collagenase, 1 mg/mL dispase, 100 U/mL DNase, 0.2% DMSO in hepatocyte basal medium) at 37°C for 35-40 min, with gentle pipetting during digestion to aid dissociation and reduce clumping. After digestion, cells were collected by adding Versene and centrifuged at 400 x g for 5 min at RT. The pellet was resuspended in Versene and further incubated at 37°C for 45 min to ensure the majority of cells were dissociated into single cells. Single-cell suspensions were blocked in 1% BSA on ice for 1 h, then stained with mouse anti-ASGR1 antibody (BD Pharmingen, #563654), followed by incubation with anti-mouse IgG microbeads (Milenyi Biotec, #130-048-401). Magnetic separation at 4°C was used to isolate HEPs from the magnetically retained fraction. HSCs were collected from the flow-through fraction, with an optional second round of hepatocyte depletion to increase purity. Purified HEPs and HSCs were then used for downstream analyses.

Quantification of HSC activation
The purified HSCs were washed with DPBS twice and directly lysed in 2 x western blot lysis buffer. The expression of intracellular activation markers were analyzed using indicated antibodies.

Triacylglycerol (TAG) assay
Intracellular triacylglycerol levels in hPSC-derived HEPs treated with purified exosomes were quantified using a commercial kit (Promega, #J3160) following the manufacturer's instructions.

ELISA
To assess the secretion of human cytokines TGFβ1, IL6, and IL1β in liver cultures treated with exosomes, culture supernatants were collected at the indicated time points, and cytokine concentrations were determined using ELISA kits (R&D Systems, #DY240, #DY206, #DY201) according to the manufacturers' instructions. For quantification of albumin secretion, liver cultures were washed with DMEM/F12 medium and incubated in albumin-free medium for 3 h, after which the supernatants were analyzed by a ELISA kit (R&D Systems, #DY1455) according to the manufacturers' instructions.

Collagen quantification.
Intracellular collagen content in HSCs from different treatments was measured using a colorimetric hydroxyproline assay kit (Sigma-Aldrich, #MAK569) following the manufacturer's instructions.

RNA extraction and quantitative RT-PCR
Total RNA was isolated from tissues or cells by Quick-RNA Miniprep Kit (Zymo Research, #R1055) and used for RT-PCR (Bio-Rad, #1708840) according to the manufacturer's protocol.

To quantify the viral copies in mice tissues, the complementary DNA was used for qPCR analysis using Bio-Rad CFX Connect 96-well Real-Time qPCR module system with PrimeTime Gene Expression Master Mix (IDT, #1055770). The 2019-nCoV RUO Kit (IDT, #10006713) was used to measure SARS-CoV-2 viral RNA. Viral load was determined by 2019-nCoV_N_Positive Control (IDT, #10006625).

For quantification for other gene transcripts, qScript^{TM} One-Step qRT-PCR Kit (Quanta Biosciences, #95057-050) on CFX96 real-time PCR system (Bio-Rad) were used following the manufacturer's protocol. Primer sequences are described in the following table.

Primer sequences used for RT-PCR		
Genes	Forward primers	Reverse primers
Pparg (mouse)	GCCCTTTACCACAGTTGATTTCT	GTGATTTGTCCGTTGTCTTTCCT

Il-1β (mouse)	TGGACCTTCCAGGATGAGGACA	GTTCATCTCGGAGCCTGTAGTG
Il-6 (mouse)	GAACAACGATGATGCACTTGC	TCCAGGTAGCTATGGTACTCC
Il-10 (mouse)	CTTACTGACTGGCATGAGGATCA	GCAGCTCTAGGAGCATGTGG
Tgfb1 (mouse)	CACCATCCATGACATGAACC	TGGTTGTAGAGGGCAAGGAC
Tnfα (mouse)	AATGGCCTCCCTCTCATCAGTT	CCACTTGGTGGTTTGCTACGA
ACACA (human)	CATGCGGTCTATCCGTAGGTG	GTGTGACCATGACAACGAATCT
SCD (human)	TCTAGCTCCTATACCACCACCA	TCGTCTCCAACTTATCTCCTCC
IL1B (human)	ATGATGGCTTATTACAGTGGCAA	GTCGGAGATTCGTAGCTGGA
IL6 (human)	AACCTGAACCTTCCAAAGATGG	TCTGGCTTGTTCCTCACTACT
TNF (human)	CCTCTCTCTAATCAGCCCTCTG	GAGGACCTGGGAGTAGATGAG
CCL5 (human)	CCAGCAGTCGTCTTTGTCAC	CTCTGGGTTGGCACACACTT
CXCL9 (human)	CCAGTAGTGAGAAAGGGTCGC	AGGGCTTGGGGCAAATTGTT
IL10 (human)	GACTTTAAGGGTTACCTGGGTTG	TCACATGCGCCTTGATGTCTG
CCL17 (human)	TTCTCTGCAGCACATCCACGCA	CTGGAGCAGTCCTCAGATGTCT
CXCL13 (human)	GCTTGAGGTGTAGATGTGTCC	CCCACGGGGCAAGATTTGAA
GAPDH (human)	GGAGCGAGATCCCTCCAAAAT	GGCTGTTGTCATACTTCTCATGG

Quantification and Statistical Analysis

All data were analyzed using GraphPad Prism. Continuous variables were present as Mean ± SD or median [IQR], and categorical variables were present as frequencies and percentage. Comparisons between two groups were performed using Student's t-test, while differences among multiple groups were assessed by one-way analysis of variance (ANOVA) followed by Tukey's post hoc test. *P*-values of less than 0.05 were considered significant.
